# A Bayesian Network Model for Reducing Accident Rates of Electrical and Mechanical (E&M) Work

**DOI:** 10.3390/ijerph15112496

**Published:** 2018-11-08

**Authors:** Albert P. C. Chan, Francis K. W. Wong, Carol K. H. Hon, Tracy N. Y. Choi

**Affiliations:** 1 Department of Building and Real Estate, The Hong Kong Polytechnic University, Hung Hom, Kowloon, Hong Kong, China; albert.chan@polyu.edu.hk (A.P.C.C.); franciswong@hkic.edu.hk or francis.wong@polyu.edu.hk (F.K.W.W.); 2 School of Civil Engineering and Built Environment, Queensland University of Technology, 2 George St., Brisbane, QLD 4001, Australia; carol.hon@qut.edu.au

**Keywords:** electrical and mechanical (E&M) works, accident analysis, Bayesian Networks, safety management

## Abstract

Accidents in Repair, Maintenance, Alteration, and Addition (RMAA) work have become a growing concern, in recent years. The repair and maintenance works of electrical and mechanical (E&M) installations involves a variety of trades, a large number of practitioners and a series of high-risk activities. The uniqueness of E&M work, in the RMAA sector, requires a discrete and specific research to improve its safety performance. Understanding the causal relationships between safety factors and the number of accidents becomes crucial to develop a more effective safety management strategy. The Bayesian Network (BN) model is proposed to establish a probabilistic relational network between the causal factors, including both safety climate factors and personal experience factors that have influences on the number of accidents related to E&M RMAA work. The data were collected using a survey questionnaire, involving a hundred and fifty-five E&M practitioners. The BN results demonstrated that safety attitude and safety procedures were the most important factors to reduce the number of accidents. The proposed BN provides the ability to find out the most effective strategy with the best utilization of resources, to reduce the chance of a high number of E&M accidents, by controlling a single factor or simultaneously controlling, both, the safety climate and personal factors, to improve safety performance.

## 1. Introduction

In recent years, there has been a rising trend in industrial accidents related to Repair, Maintenance, Alteration, and Addition (RMAA) work [1]. With the effect of ageing buildings and the implementation of Mandatory Building Inspection Scheme (MBIS) and Mandatary Window Inspection Scheme (MWIS), of buildings and windows, by the Buildings Department of the Hong Kong Special Administrative Region (HKSAR) Government, the volume of RMAA work are predicted to grow further. RMAA work involves a range of high-risk processes, such as work at height, at truss-out scaffolding or bamboo scaffolding, electrical works, lifting operations, falsework, and confined space work. According to the industry performance report published by Construction Industry Council [2], RMAA had a significant number of fatalities among the whole construction industry. Poor safety performance in RMAA projects are not unique to Hong Kong construction industry alone. This situation is a worldwide issue. The rising awareness about improving the energy efficiencies of existing buildings, in some countries, such as Australia, the United Kingdom, and the United States, boosts the volume of the RMAA work. The building ageing problem in developed countries is another major reason for the expansion of the RMAA work, in the construction market.

Safety is a crucial issue for RMAA projects. In 2016, the gross value of RMAA has accounted for over 54% of the whole construction industry in Hong Kong [3]. Special RMAA trades, such as electrical wiring, ventilation, water fitting maintenance, contributed to 61% of the total gross value of RMAA work. In Hong Kong, over 80% of special RMAA trades were conducted by subcontractors [4]. To gain the benefits of specialist expertise and economic flexibility, for principal contractors under a highly competitive environment, trade subcontracting is a common phenomenon in the construction industry, especially for RMAA work, in Australia [5,6]. Most of the construction projects, regardless of it being a new work or an RMAA one, are performed by a variety of subcontractors, while the main contractor tends to focus on the coordination and management. Generally, the scales of subcontracting firms are small, with limited resources. The bottom-tiers subcontractors are often contracted with unreasonably low bids. In order to make a profit to survive, these bottom tiers may sacrifice the work quality and safety [7]. Some previous studies further supported that subcontracting practices create adverse effect on health and safety, in the construction industry [4,8,9]. Retrospective evidence from related research demonstrated that the adverse influences of subcontracting on health and safety, in the construction industry, is serious across different countries in the world, such as the United Kingdom [10,11], the United States [12], Spain [13], China [14], Hong Kong [4,15], and Singapore [16].

The accident factors of RMAA work have been investigated by a few studies. Chan et al. [17], conducted a comprehensive analysis of the fatality cases, to examine the causes of fall accidents in repair and maintenance works, for residential buildings. Based on the analysis results of case studies, five strategies were suggested to prevent recurrence of similar accidents, (1) provide and maintain a safe system of work; (2) provide a suitable working platform; (3) provide adequate safety information, training, instruction, and supervision to the frontline workers; (4) provide suitable fall arresting system; and (5) maintain safe workplace. A key aspect missing from these studies was a lack of specific focus on electrical and mechanical (E&M) work in the RMAA projects. To bridge this research gap, the current research aims to explore the influence of different factors and their impact on E&M RMAA work safety, with an ultimate goal of reducing the accident rate of electrical and mechanical (E&M) work. Electrical and mechanical (E&M) work consists of five key trades, i.e., lift, fire services, plumping and drainage, air-conditioning, and electrical wiring. E&M work are considered as high-risk activities because of the unique work features. The major hazards are activities that involve electrical wiring, electrical hazard at switch gear works, lifting of chillers and generators, machinery for lifts and escalators, confined working spaces for pumping and drainage work, large amount of welding, and using handheld tools. Electrical hazards are regarded as the most significant threat to E&M workers. Accidents related to electrical work cause serious injuries to the frontline workers. Some previous safety researches demonstrated that electrical hazards are regarded as one of the major causes of construction fatality in the United States [18] and Taiwan [19].

This paper adopts a systemic decision approach, based on the Bayesian network (BN) to provide a powerful tool for reasoning in a dynamic and complex project environment. This approach can accurately illustrate the effect of each factor on the occurrence of E&M accidents. The proposed approach can be used as a decision-making tool by practitioners in the industry to compare various intervention strategies and determine the best strategies among them.

## 2. Research on Bayesian Networks

The Bayesian Network (BNs) is an effective tool to demonstrate complicated relationships. Based on the theorem by Thomas Bayes [20], it is a powerful tool for knowledge representation and reasoning, to visually present the probabilistic relationships among a set of variables [21,22]. The application of BNs is suitable for small and incomplete data sets. It is particularly convenient to model causal relationships among variables (with a combination of different sources of information), handling of uncertainty for decision analysis, predicting the effectiveness and outcomes of various strategies, and selecting the best from them [23,24]. BNs have been used extensively to develop decision-support systems in a medical diagnosis, risk assessments and management, and in ecosystem and environmental management [25,26,27]. In recent years, the BN has been increasingly adopted in construction-related research. It has been applied in various disciplines, for example, project cost control and forecasting [28,29], risk analysis in construction projects [30,31], structural health monitoring of buildings [32,33], accident analysis and safety control [34,35], and labor productivity [36]. Most of the existing safety research adopted conventional statistical methods, such as factor analysis, ANOVA, and multiple regression [37,38,39]. While these methods are useful in identifying the relationship between the accident and each of its causal variables, they neglect the interdependencies of the causal factors [40]; an accident is often the combined outcome of a number of factors. These methods have a limited ability in revealing complex cause–effect relationships and constructing a model for predicting accidents [41]. Structural Equation Model (SEM) techniques have been developed to learn causal relationships from data [42]. SEM has been adopted in some previous studies for modeling safety factors and occupational accidents [43,44], SEM is suitable for prediction performance of linear relationships. When relationships are non-linear, a poor prediction performance will be resulted. The Bayesian Network is a generative statistical model representing a class of joint probability distributions. Comparing the applications of these two techniques, SEM is mostly used for identifying what factors determine the value of this variable, whereas the BN is adopted to calculate the probability of other variable (child node) changes, when the parent nodes were intervened. BNs have become increasingly popular for safety research, recently. Zhou et al. [45] proposed a BN model to demonstrate the influences of safety climate factors and personal experience factors, on the safety behavior of workers in construction. Mohammadfam et al. [46] provided a BN model for managing and improving safety behavior of employees, for construction projects in Iran. Nguyen et al. [34] developed a generic Bayesian Networks model to predict the safety risk of working at heights. The results provide probabilities associated with different states of safety risk. It allows the industrial practitioners to identify appropriate preventive measures and safety strategies to reduce the risk of fall. Zhang et al. [47] presented a novel and systematic decision support model for the safety control of complex project environments, based on the BN. It can be used by the industrial practitioners to increase the likelihood of a successful project, in complex environments. Bayesian networks are useful in fields where there is a need for prediction and the outcome is uncertain. They are extensively used in diagnosing, managing risk, modeling, monitoring, and forecasting. With the help of BNs, quantifiable and justifiable decisions can be made. This research provides an opportunity to illustrate the application of decision support system for safety personnel and the management staff, to formulate suitable strategies to reduce E&M accident risk.

## 3. Methodology

**Figure 1:**
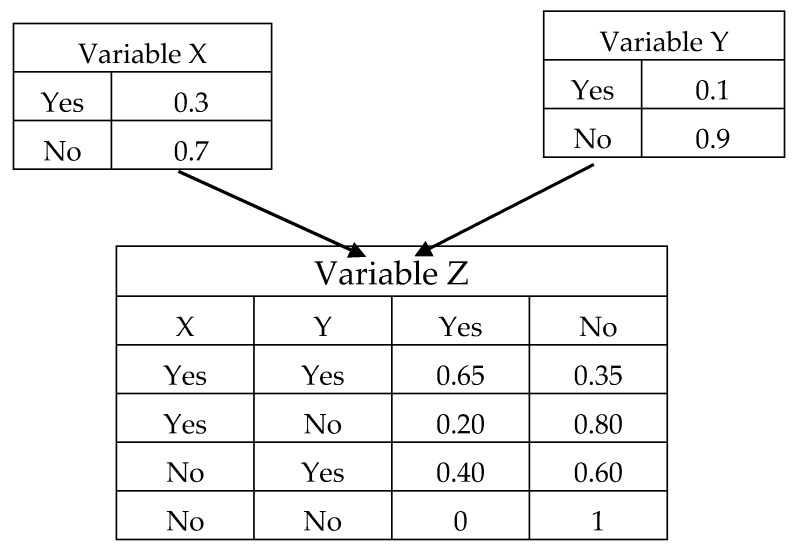
An example of a Bayesian Network structure and the Conditional Probability Tables (CPT).

It is well known that the effects of direct or indirect factors on the number of accidents, are very complicated. As stated in the previous section, Bayesian Networks (BNs) are regarded as flexible and powerful techniques for graphically modelling the causal interrelationships among some variables and quantitatively representing this intricate relationship [48,49]. A BN basically consists of two parts—a quantitative and a qualitative part. The qualitative part is regarded as structure learning, which aims to establish the structure of a directed acyclic graph (DAG) which is composed of a set of nodes and directed edges (arrows). When two nodes are connected by an edge, the node with a link directed to a subsequent node is called a “parent node” of the subsequent node. The subsequent node is called a “child node”. Child nodes are conditionally dependent on their parent nodes. For instance, nodes X and Y are the parent nodes of node Z (Figure 1). As a BN is a DAG, a cycle cannot be formed among the variables within the network.

The quantitative part of a BN, which is called parameter learning, aims to determine the conditional probability distribution of each node, according to the established BN structure. This part is based on the conditional probability theory or Bayes’ theorem. Bayes’ theorem which also known as Bayes’ rule or Bayes’ law can be expressed as the following formula:

$$P\left(X|Y\right)=\frac{P\left(Y|X\right)P\left(X\right)}{P\left(Y\right)}$$

It describes the probability of an event, where P(X│Y) is the likelihood of event X, given that Y occurs; P(Y│X) represents the likelihood of event Y given that X occurs; P(X) and P(Y) are the probabilities of X and Y occurring independently. The conditional probability tables (CPTs) show the probability of various states of a node, in accordance with the configuration of its parent states. Figure 1 illustrates the state and the conditional probability tables of variables Z. The Netica software package, developed by Norsys Software Corporation was adopted in this study. The developed BN can be updated when new information becomes available.

## 4. Questionnaire Survey

An extensive questionnaire survey of the RMAA work of E&M practitioners, in Hong Kong, was conducted to collect data for quantitatively establishing the relationship of the BN. With the purpose of investigating the relationship of the safety climate and the personal factors, on the number of E&M accidents, all survey respondents were requested to complete a safety climate questionnaire. The questionnaire consisted of three parts. The first part included thirteen questions related to demographic and personal information, i.e., (a) working level, (b) work trade, (c) age, (d) gender, (e) marital status, (f), number of family members supported by the respondent, (g) education level, (h) direct employer, (i) length of services, (j) years of working experience, (k) safety training, (l) smoking habit, and (m) drinking habit. The second part of the questionnaire survey adopted thirty-eight questions from the Safety Climate Index (SCI) survey of the Occupational Safety and Health Council (OSHC) of Hong Kong [50], to measure safety climate. The Safety Climate Index (SCI) survey is a tool to measure safety climate of an organization. It is designed to suit the local context of the construction industry in Hong Kong. With the prior corroboration of the Occupational Safety and Health Council Hong Kong (OSHC), related government work departments, private property developers, and major contractors, the reliability and practicality of the tool for measuring safety climate in the construction industry, have been validated. The thirty-eight SCI survey items were rated on a five-point Likert scale, from ‘1’ representing ‘strongly disagree’ to ‘5’ representing ‘strongly agree’. The third part incorporated four questions to measure the number of accidents met by the survey respondents, in the past twelve months. The four questions asked the respondents to indicate the number of near-miss incidents, accident or injuries not requiring absence from work, accident or injuries requiring absence from work not exceeding three consecutive days, and injuries requiring absence from work exceeding three consecutive days, in the past twelve months, respectively. These questions reflected the frequency and the severity of accidents suffered by the worker. The pilot questionnaire was reviewed by thirteen experts who have acquired over fifteen years of experience in the industry, before the large-scale survey. The background information of the thirteen experts has been summarized in Table 1. This process was essential to ensure the research fully met the needs and concerns of the RMAA sector, in the construction industry. With the support of a large E&M engineering company in Hong Kong, the questionnaire survey was administered to its direct labor and the subcontractors, face-to-face in a meeting room, before they headed off to on-site work, in the morning. To enhance the quality of the responses, fourteen trained student helpers were recruited to assist the research participants to answer the questionnaire. As a result, more than seven hundred sets of the completed questionnaire were received, in the four months. In this research, respondents not in E&M trades or without hand-on experience in E&M RMAA work, was excluded. Among them, a total of a hundred and fifty-five sets of questionnaires that were completed by E&M practitioners, were extracted for the analyses in this study.

**Figure 2:**
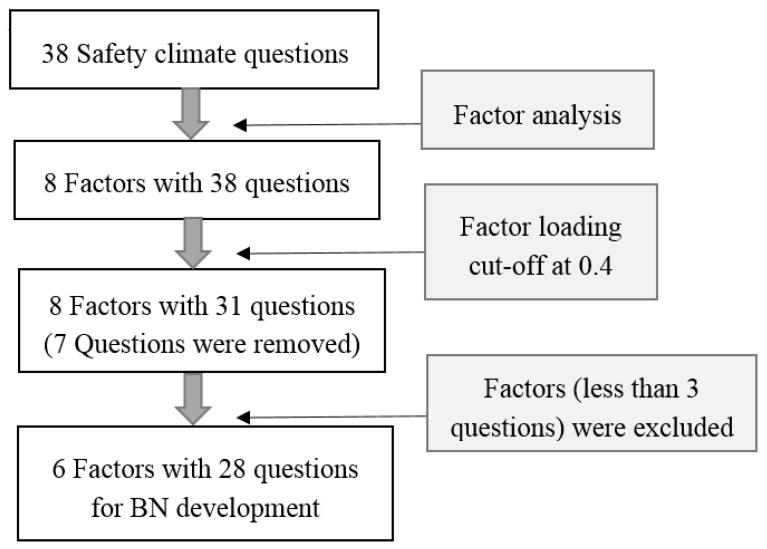
The process of factor analysis. Notes: Cronbach’s α test was used to measure the internal consistency of each factors.

For developing the structure of the BN, the thirty-eight safety climate questions (items) in the survey form were reduced and grouped into six factors, through a factor analysis with promax rotation, and an internal consistency test was carried out (Figure 2). There are two ways to rotate factors—oblique and orthogonal. An orthogonal rotation method, such as equamax, quartimax, and varimax, requires the factors to be independent of each other, while an oblique rotation method (i.e., promax, direct oblimin, etc.) is suitable for factors that are correlated [51]. The results of an orthogonal rotation may produce misleading results with the presence of significant correlations among factors. In safety research, we generally expect some correlation among the factors. The oblique rotation approach would be appropriate to obtain several theoretically meaningful factors. Promax is one of the most commonly used oblique rotation methods which has been adopted by a number of researchers [52,53,54]. Therefore, the promax rotation method was finally applied to this study.

In the study, eight safety climate factors were identified from the thirty-eight safety climate items, with a total variance of 59.6%. Based on the factor analysis results, items which had a factor loading of less than 0.4, in the factor group, were removed [45,55]. Seven items which had a factor loading of less than 0.4 were excluded and eight factors with thirty-one items remained. Subsequently, factors with less than three items were excluded [56,57]. As suggested by Harman [58], to ensure sufficient loadings of each factor, each factor must contain at least three components to define a factor. This brought about a further removal of two factors. Cronbach’s α test was used to measure the internal consistency of the questionnaire results. In this study, the calculated Cronbach’s α was 0.786, which was higher than the generally-accepted, minimum, desired value of 0.7 [59]. To further examine the internal consistencies of each factor, the Cronbach’s α for the six defined safety climate factors, with the five-point Likert scales, was calculated. The Cronbach’s α of all six factors were higher than the threshold value of 0.7, which indicated that the internal consistency of each factor was adequate for further analysis. As a result, the six safety climate factors with twenty-eight items were used for the development of the BN, in the next stage. The six factors were: (1) Safety attitudes, (2) understanding of work risks, (3) management commitments, (4) safety resources and equipment, (5) safety procedures, and (6) workmate influences. Questions pertaining to the corresponding safety climate factor were rated on a five-point Likert scale.

## 5. Bayesian Network—Structure Learning

**Figure 3:**
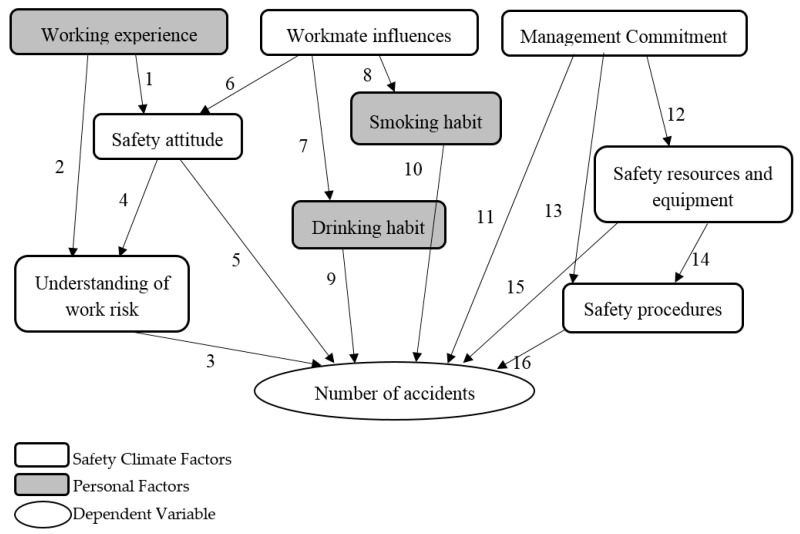
The established Bayesian Network structure.

Some cross-relationships between safety climate factor and personal factors were present in the BN structure. Zhou et al. [45] advocated that workmate influences would affect safety attitudes, smoking habit, and drinking habit. The research of Meliá and Becerril [60] pointed out that workers’ behavior or actions such as smoking or alcohol consumption habit is a significant factor of accidents. The unhealthy behavior of workers was highly influential among workmates. Similarly, management commitments were believed to alter the safety resources and equipment, safety procedures, and number of accidents [45,61,62]. Flin et al. [63] advocated that management commitment has a great influence on the allocation of safety resources and facilitation of safety polices and safety equipment. The working experience factor may affect the understanding of work risk and safety attitude of workers [44,64]. In addition to the six safety climate factors identified in the previous stage, three personal factors, i.e., working experience, drinking habit, and smoking habit were, therefore, included in the BN model. Details of the BN constructs are provided in Table 2. The structure of the BN is shown in Figure 3. It was constructed based on several safety models reported in previous studies [45,65,66]. Literature supporting the causal relationship of the identified factors are summarized in Table 3.

## 6. Bayesian Network—Parameter Learning

After establishing the BN structure, conditional probabilities of the variables were determined on the basis of the data input from the survey respondents. The notion of conditional probability in BN is particularly useful, as this concept facilitates the real-world situation that the probability of a child event is conditional on the probability of its parent events. The conditional probabilities are often represented in conditional probability tables (CPTs). The data are originally collected as a five-point Likert scale, through the questionnaire. If each node is designated by five states, the conditional probabilities table of the node would be very huge and would require quite large samples and an exhaustive computational effort. To avoid this issue, Zhou et al. [45] and Jitwasinkul et al. [35] suggested (and adopted) a three-point Likert scale that can be reformed, based on the data originally collected from a five-point Likert scale questionnaire. The five level of agreement were integrated into three states, as follows:

(1) States of “strongly agree” and “agree” are combined into one new state of “(S1) Good”,
(2) the state of “neither agree nor disagree” is changed to be “(S2) Average”, and
(3) the states of “strongly disagree” and “disagree” are integrated as “(S3) poor”.

## 7. Bayesian Network Analysis Results

The items related to each safety climate factor have been summarized in Table 2. The values of each state of the nodes, equal the average scores of the relevant items with the same weight. For example, the node “safety attitudes” consisted of four items, the value of each state (S1, S2, & S3) is calculated based on the average score of the three questions listed in Table 2. The values of the other nodes were computed in the same way.

### 7.1. Conditional Probability Table (CPT)

As stated previously, the relationships of variables in a BN model are quantified by a set of conditional probabilities and are summarized in CPTs. It generally shows the probability of all states of a specific child node, under all possible combinations of the states of its parents. For instances, the CPT of the “safety attitude” node was calculated, based on the survey data, as shown in Table 4. The parent nodes of “safety attitude” were “working experience” and “workmate influences”. There were nine possible state combinations including:

(1) <“working experience” = Short, “workmate influences” = Positive>,
(2) <“working experience” = Short, “workmate influences” = Neutral>,
(3) <“working experience” = Short, “workmate influences” = Negative>,
(4) <“working experience” = Medium, “workmate influences” = Positive>,
(5) <“working experience” = Medium, “workmate influences” = Neutral>,
(6) <“working experience” = Medium, “workmate influences” = Negative>,
(7) <“working experience” = Long, “workmate influences” = Positive>,
(8) <“working experience” = Long, “workmate influences” = Neutral>,
(9) <“working experience” = Long, “workmate influences” = Negative>,

The sum of the probabilities of “safety attitude”, in each row of Table 4, should equal to one.

**Figure 4:**
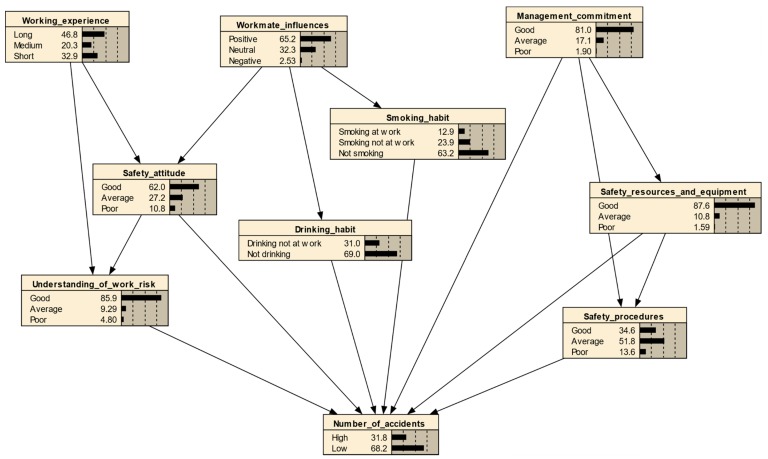
Bayesian network structured to analyze number of E&M work-related accidents.

On the basis of data obtained from the questionnaire survey, the probabilities of each node and states are shown in Figure 4. For the node “safety attitude”, 62% of respondents considered themselves as possessing “good” safety attitude, while 27.2% and 10.8% of them considered themselves having “average” and “bad” safety attitudes, respectively. The total probability of all states, at the nodes, should be 100%. Regarding the node “working experience”, 46.8% of respondents gained “long (i.e., over 10 years)” period of working experience, 20.3% and 32.9% of them had “medium (6 to 10 years)” and “short (5 or less than 5 years)” periods of working experience.

### 7.2. Bayesian Prediction for E&M RMAA Accidents

Bayesian inference is an essential technique among the various applications of Bayes’ theorem. The established BN can be applied for the predictive reasoning of E&M RMAA accidents. In this process, the most effective safety strategies to reduce the number of accidents in the industry can be identified. The constructed BN is shown in Figure 3. Based on the computed probabilities of all factors, the probability of a “high” number of accidents occurring, was 31.8%, whereas, the probability of a “low” number of accidents occurring, was 68.2% (Figure 3).

### 7.3. Single Strategy to Reduce the Number of Accidents

Probability of the outcome node highly relies on all of the factors (i.e., node) mentioned above [81]. Safety management strategies can be evaluated on the basis of the outcome node and the number of accidents, by controlling the different factors. Different factors may have distinctive levels of influence on the number of accidents that occurred. Sensitivity analysis can be performed by the computational program to measure how changes of safety factors (e.g., safety procedures, safety attitude, and safety resources and equipment, etc.) affect the number of accidents. By comparing the sensitivity of the number of accidents, an optional strategy can be identified [45]. This analysis is effective for decision-making support for management teams, to reduce accidents [32,35,82]. The results of sensitivity analysis of the nine factors, on the number of accidents, are summarized in Table 5. It demonstrated that “safety attitude” has the most effective influence on the probability of “high” number of accidents. The probability of “high” number of accidents decreased from 31.8% to 27.8%, when the node “safety attitude” changed from “average” or “bad” to “good”. This revealed that when safety attitude of workers is improved, efficiently, the likelihood of a “high” number of accidents (i.e., suffered two or more accidents and occupational injuries in the past twelve months) would be greatly decreased. The predominant effect of safety attitude on safety performance in the construction industry has been highlighted by some previous research. For example, Wu et al. [30] demonstrated a positive effect of safety attitude on safety performance. The research of Teo et al. [64] pointed out that there is a positive link between safety performance and workers’ attitudes. Accidents occur as a result of poor attitudes of workers, which are difficult to control and monitor.

According to Table 5, “Safety procedures” and “Management commitment” were considered as the second and the third most effective factors to reduce the probability of a “high” number of accidents. The probability of a “high” number of accidents decreased by 3.9% and 3.1%, respectively. It has been reinforced by previous studies that safety procedures and management commitment are two prime controls to reduce occupational accidents [83,84,85]. The survey results conducted by Abudayyeh et al. [85] presented a clear statistical correlation between the management’s commitment and accident rates. E&M work involved a substantial amount of high-risk activities, such as electrical works, works at height, works with machinery, and works at confined spaces, etc. When hazards cannot be cut off with physical barriers, safety procedures (e.g., lockout–tagout devices, or air testing in confined spaces) are important to prevent exposure of workers to the hazards. A clear management commitment might also positively influence the safety culture, monitoring and improvement mechanisms, and employee involvement, so that the likelihood of accident occurrence in RMAA E&M work can be reduced.

The results of the sensitivity analysis also showed that “working experience” has the least effect on reducing the number of accidents. The probability of “high” number of accidents only decreased by 0.4%, when the node “working experience” changed from “short” to “long”. Although some previous studies [68,73] indicated that young workers with less experience are more prone to accidents, there is no strong evidence to support that the number of accidents would be reduced with the increase in the workers’ years of experience. Based on the sensitivity analysis, the mutual effect of the workers’ length of working experience on safety performance, is in doubt.

### 7.4. Joint Strategies to Reduce the Number of Accidents

The previous sensitivity analysis of a single strategy demonstrated that “safety attitude” is the most effective factor to reduce the occurrence of a “high” number of accidents. In this stage, the sensitivity tests are further conducted to assess the effectiveness of different combinations of the two-factor strategies. As shown in Table 6, improving both “safety attitude” and “safety procedures” is the best two-factor strategy to reduce the number of accidents. It is supported by previous studies that safety attitude and safety procedures are the most important elements to achieve a better safety performance [37,62,68]. The work environment in an E&M work is generally more hazardous than general building work, due to the use of dangerous working processes and equipment, hazardous materials, and complex working procedures, all of which increases the potential for serious accidents and injuries [19]. RMAA work construction generally lasts for a short construction period with less safety resources, equipment, and inadequate safety supervision. The safety attitude and safety awareness of RMAA workers are poor, in general. If safety attitude of workers is poor, they may not be willing to follow the safety procedures, strictly. Thus, it is not surprising that the improvement of both factors would substantially reduce the probability of a “high” number of accidents.

**Figure 5:**
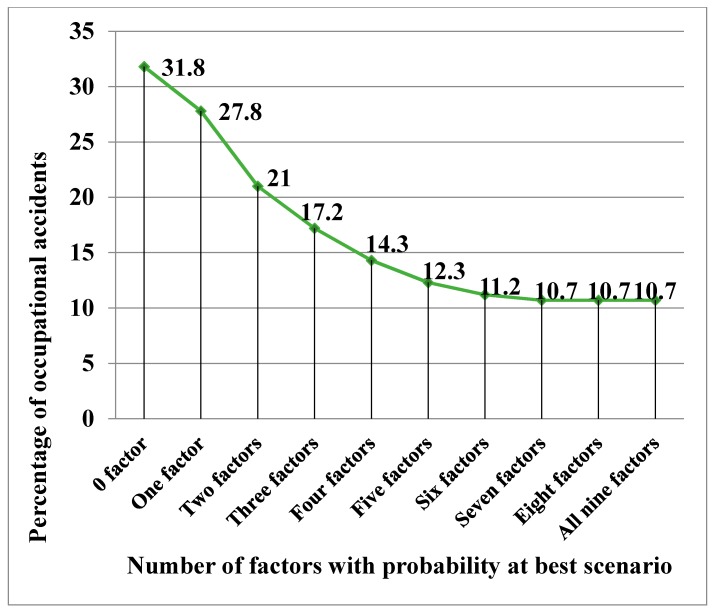
Sensitivity analysis results of single and joint-factor strategies, from two to nine factors.

“Management commitment” was another factor that had a tremendous power to reduce the number of accidents. As illustrated in Figure 5 and Table 7, the “high” number of accidents further dropped from 21% to 17.2%, when three factors were improved to a “good” condition. The sensitivity analysis results of the single and joint-factor strategies, from two to nine factors, are summarized in Table 7.

### 7.5. Diminishing Returns of Improving the Safety Factors

A trend of diminishing returns on safety investment was observed in Figure 5. This implies that the investment of improving the safety factors does not necessarily reduce a “high” number of accidents to the same level. At this point, after improving the seven safety factors to a “good” condition, no further reduction in a “high” number of accidents can be achieved. Therefore, there is no point in deploying further resources to improve the factor of “workmate influence” and “working experience” for accident reduction. If resource is unlimited, one can combine all factors to yield maximum improvement. However, in case of a limited resource, one can apply this analysis to evaluate the effectiveness of each strategy and determine the best combination of strategies, in order to yield the optimal outcome [31,35,46]. The method is a practical and easily-implemented technique to identify the most effective strategies for reducing E&M RMAA accidents.

## 8. Limitations and Area for Future Research

It is acknowledged that the design of the questionnaire survey has some limitations. The rating of safety climate and safety performance of workers relied heavily on self-reported measures. The BN was constructed on the basis of two categories of factors, six safety climate factors, and three personal factors. The thirty-eight safety climate items were grouped into six factors by factor analysis. However, the three personal factors, although intuitively believed to be influential to safety performance, were applied into the BN, without conducting a factor analysis on them. All the factors should, ideally, be grouped and extracted through the factor analysis. Further research could be conducted in other developed cities to group and extract all these factors, through factor analysis, for intriguing comparisons. In addition, Resilience Engineering (RE), an effective tool for risk management with the consideration of an inherent complexity of system [86], can be adopted for E&M RMAA research. Safety and risk management research works involve in-depth investigation of how systems perform, and the interactions between agents, rather than individual agents [87]. RE has been regarded as a safety management paradigm that is suited to the nature of complex socio-technical systems (CSTSs). Thus, RE can be adopted in further investigations of the complex activities in the realm of E&M RMAA safety.

## 9. Conclusions

E&M work are one of the most hazardous and complex trades in the RMAA sector, which demands for a comprehensive research to enhance the safety performance. Investigation of the causal relationship between safety factors and the number of accidents is important, in order to develop more effective safety strategies. The major objective of this research was to develop a generic BN model to reduce the accident rates of E&M RMAA work. This is an effective decision support tool, which enables the industrial practitioners to identify and prioritize the best strategies for reducing E&M accidents. The structural BN between different safety factors was established in this study. The probability of each factor was quantified, based on the feedback of a hundred and fifty-five E&M RMAA practitioners. Respondents who suffered two or more accidents and occupational injuries in the past twelve months were categorized as having a “high” number of accidents. The results of the BN modeling found that the probability of having a “high” number of accidents was 31.8%. Through a sensitivity analysis using a BN, the most influential factor for reducing a “high” number of accidents was determined. The results showed that “safety attitude” was the most influential factor for reducing a “high” number of accidents (from 31.8% to 27.8%). A trend of diminishing returns on the safety investment was observed. This implied that the investment of improving the safety factors does not necessarily reduce the “high” number of accidents to the same level. If resource is unlimited, one can combine all factors to yield the maximum possible improvement. However, in case of a limited resource, one can evaluate the effectiveness of each strategy and determine the best combination of strategies in order to yield the optimal outcome by the BN modeling and sensitivity analysis. This research results vividly demonstrate how the BN modeling and sensitivity analysis can help identify the best strategies for reducing E&M RMAA accidents. Similar techniques can be applied to other types of projects and to other sectors, respectively.

## Figures and Tables

**Table 1:** Background information of the thirteen experts.

	Organization/Trades	Position
1	Contractor	Safety Manager
2	Contractor	Manager
3	Property management company	Technical Manager
4	Hong Kong government	Deputy Chief Occupational Safety Officer
5	Hong Kong government	Senior Manager (Safety and Health)
6	Hong Kong government	Senior Structural Engineer
7	Self-regulatory body of insurers	Representative
8	Quasi-government body	General Manager
9	Occupational Safety and Health Council	Principle Consultant
10	Construction Industry Institute—Hong Kong	Director
11	Private developer	Manager
12	Electrical and mechanical contractor	Executive Director
13	Utility service company	Safety, Health, Environment and Quality Manager

**Table 2:** Constructs of the Bayesian Network (BN).

Category	Factor	Question
Safety Climate Factors	Safety attitudes	Accident investigations are mainly used to identify who should be blamed People are just unlucky when they suffer from an accident Little is done to prevent accidents until someone gets injured People here always work safely even when they are not being supervised
	Understanding of work risks	I fully understand the health and safety risks associated with the work for which I am responsible I am clear about what my responsibilities are for health and safety Work health and safety is not my concern
	Management commitments	All the people who work in my team are fully committed to health and safety The company encourages suggestions on how to improve health and safety There is good preparedness for emergency here My immediate boss often talks to me about health and safety matters on site Staff are praised for working safely
	Safety resources and equipment	People can always get the equipment which is needed to work according to the health and safety procedures People here always wear their personal protective equipment (e.g., eye protectors, masks, ear protectors, etc.) when they are supposed to There are always enough people available to get the job done
	Safety procedures	I feel involved in the development and review of health and safety procedures or conduct risk assessment Some health and safety rules or procedures do not reflect how the job is now done Some health and safety rules or procedures are difficult to follow Health and safety procedures are much too stringent in relation to the risks Some jobs here are difficult to do safely Not all the health and safety rules or procedures are strictly followed here Supervisors sometimes turn a blind eye to people who are not observing the health and safety procedures Accidents which happened here are always reported I know that if I follow the safety rules or procedures, I will not get hurt
	Workmate influences	It is important for me to work safely if I want to keep the respect of others in my team Time pressures for completing jobs are reasonable My workmates would react strongly against people who break health and safety procedures Some of the workforces pay little attention to health and safety
Personal Factors	Working experience	Long—over 10 years working experience Medium—6 to 10 years working experience Short—5 or less than 5 years working experience
	Smoking habit	Smoking at work Smoking, but not at work Not smoking
	Drinking habit	Drinking at work Drinking, but not at work Not drinking
Dependent Variable	Number of accidents	High—Suffered two or more accidents and occupational injuries in the past 12 months Low—Suffered only one or no accident or occupational injury in the past 12 months

**Table 3:** Literature supporting the causal relationship of the identified factors.

No.	Relationship Pairs	References
1	Working experience & Safety attitude	[44,64,67]
2	Working experience & Understanding of work risk	[45,68]
3	Understanding of work risk & Number of accidents	[69,70]
4	Safety attitude & Understanding of work risk	[71]
5	Safety attitude & Number of accidents	[44,68]
6	Workmate influences & Safety attitude	[45,72,73,74]
7	Workmate influences & Drinking habit	[45,60,74]
8	Workmate influences & Smoking habit	[74]
9	Drinking habit & Number of accidents	[74,75]
10	Smoking habit & Number of accidents	[74,76,77]
11	Management Commitment & Number of accidents	[37,67]
12	Management Commitment & Safety resources and equipment	[61,78]
13	Management Commitment & Safety procedures	[45,63]
14	Safety resources and equipment & Safety procedures	[63,79,80]
15	Safety resources and equipment & Number of accidents	[61,62,67]
16	Safety procedures & Number of accidents	[62,64,67,79]

**Table 4:** Conditional probability table (CPT) of the “safety attitude” node.

“Safety Attitude”			Parents’ Node of “Safety Attitude”	
Good	Average	Bad	Working Experience	Workmate Influences
0.54	0.32	0.14	Short	Positive
0.45	0.48	0.07	Short	Neutral
0.34	0.33	0.33	Short	Negative
0.79	0.08	0.13	Medium	Positive
0.58	0.33	0.09	Medium	Neutral
0.50	0.25	0.25	Medium	Negative
0.69	0.20	0.10	Long	Positive
0.61	0.33	0.06	Long	Neutral
0.40	0.20	0.40	Long	Negative

**Table 5:** Sensitivity of single strategy to reduce the number of accidents.

Probability at Best Scenario of One Factor	“High” Number of Accidents		Sensitivity on Number of Accidents
	Original Value	New Value	
Safety attitude	31.8%	27.8%	4%
Safety procedures	31.8%	27.9%	3.9%
Management commitment	31.8%	28.7%	3.1%
Understanding of work risk	31.8%	28.9%	2.9%
Smoking habit	31.8%	29.2%	2.6%
Safety resources and equipment	31.8%	29.6%	2.2%
Drinking habit	31.8%	29.8%	2%
Workmate influences	31.8%	30.6%	1.2%
Working experience	31.8%	31.4%	0.4%

**Table 6:** Two-factor strategies with the highest effect to reduce the number of accidents.

Joint Strategy	“High” Number of Accidents		Sensitivity on Number of Accidents
	Original Value	New Value	
(“Safety attitude” = Good) + (“Safety procedures” = Good)	31.8%	21%	10.8%
(“Safety attitude” = Good) + (“Management commitment” = Good)	31.8%	23.8%	8%
(“Safety procedures” = Good) + (“Understanding of work risk” = Good)	31.8%	24.3%	7.5%

**Table 7:** Sensitivity analysis results of single and joint-factor strategies, from two to nine factors.

Number of Factors with Probability at Best Scenario	“High” Number of Accidents	Percentage of Improvement
0 Factor	31.80%	0%
1 Factor	27.80%	12.60%
(“Safety attitude” = Good)		
2 Factors	21%	24.50%
(“Safety attitude” = Good) +		
(“Safety procedures” = Good)		
3 Factors	17.20%	18.10%
(“Safety attitude” = Good) +		
(“Safety procedures” = Good) +		
(“Smoking habit” = Not Smoking)		
4 Factors	14.30%	16.90%
(“Safety attitude” = Good) +		
(“Safety procedures” = Good) +		
(“Smoking habit” = Not Smoking) +		
(“Understanding of work risk” = Good)		
5 Factors	12.30%	14.00%
(“Safety attitude” = Good) +		
(“Safety procedures” = Good) +		
(“Smoking habit” = Not Smoking) +		
(“Understanding of work risk” = Good) + (“Management commitment” = Good) OR		
(“Safety resources and equipment” = Good)		
6 Factors	11.20%	8.90%
(“Safety attitude” = Good) +		
(“Safety procedures” = Good) +		
(“Smoking habit” = Not Smoking) +		
(“Understanding of work risk” = Good) + (“Management commitment” = Good) +		
(“Safety resources and equipment” = Good)		
7 Factors	10.70%	4.50%
(“Safety attitude” = Good) +		
(“Safety procedures” = Good) +		
(“Smoking habit” = Not Smoking) +		
(“Understanding of work risk” = Good) + (“Management commitment” = Good) +		
(“Safety resources and equipment” = Good) +		
(“Drinking habit” = Not Drinking)		
8 Factors	10.70%	0%
(“Safety attitude” = Good) +		
(“Safety procedures” = Good) +		
(“Smoking habit” = Not Smoking) +		
(“Understanding of work risk” = Good) + (“Management commitment” = Good) +		
(“Safety resources and equipment” = Good) +		
(“Drinking habit” = Not Drinking) +		
(“Workmate influence” = Positive) OR (“Working experience” = Long”)		
9 Factors	10.70%	0%
(“Safety attitude” = Good) +		
(“Safety procedures” = Good) +		
(“Smoking habit” = Not Smoking) +		
(“Understanding of work risk” = Good) + (“Management commitment” = Good) +		
(“Safety resources and equipment” = Good) +		
(“Drinking habit” = Not Drinking) +		
(“Workmate influence” = Positive) +		
(“Working experience” = Long”)		
